# Local steroid injection for moderately severe idiopathic carpal tunnel syndrome: Protocol of a randomized double-blind placebo-controlled trial (NCT 00806871)

**DOI:** 10.1186/1471-2474-11-76

**Published:** 2010-04-21

**Authors:** Magnus Flondell, Manfred Hofer, Jonas Björk, Isam Atroshi

**Affiliations:** 1 Department of Orthopedics, Hässleholm and Kristianstad Hospitals, SE-28125 Hässleholm, Sweden; 2 Department of Clinical Sciences, Lund University, SE-22100 Lund, Sweden; 3 Department of Physiotherapy, Kristianstad Hospital, SE-29185 Kristianstad, Sweden; 4 Competence Centre for Clinical Research, Lund University Hospital, SE-22185 Lund, Sweden

## Abstract

**Background:**

Patients with idiopathic carpal tunnel syndrome (CTS) are commonly treated with steroid injection into or proximal to the carpal tunnel. However, evidence for its efficacy beyond one month has not been established in randomized placebo-controlled trials. The primary aim of this randomized trial is to assess the efficacy of steroid injection into the carpal tunnel in relieving symptoms of CTS in patients with symptoms of such severity to warrant surgical treatment but have not been treated with steroid injection.

**Methods/Design:**

The study is a randomized double-blind placebo-controlled trial. Patients referred to one orthopedic department because of CTS are screened. Eligibility criteria are age 18 to 70 years, clinical diagnosis of primary idiopathic CTS and abnormal nerve conduction tests or clinical diagnosis made independently by two orthopedic surgeons, failed treatment with wrist splinting, symptom severity of such magnitude that the patient is willing to undergo surgery, no severe sensory loss or thenar muscle atrophy, and no previous steroid injection for CTS. A total of 120 patients will be randomized to injection of 80 mg Methylprednisolone, 40 mg Methylprednisolone, or normal saline, each also containing 10 mg Lidocaine. Evaluation at baseline and at 5, 10, 24 and 52 weeks after injection includes validated questionnaires (CTS symptom severity scale, *Quick*DASH and SF-6D), adverse events, physical examination by a blinded assessor, and nerve conduction tests. The primary outcome measures are change in the CTS symptom severity score at 10 weeks and the rate of surgery at 52 weeks. The secondary outcome measures are the score change in the CTS symptom severity scale at 52 weeks, time to surgery, and change in *Quick*DASH and SF-6D scores and patient satisfaction at 10 and 52 weeks. The primary analysis will be carried out using mixed model analysis of repeated measures.

**Discussion:**

This paper describes the rationale and design of a double-blind, randomized placebo-controlled trial that aims to determine the efficacy of two different doses of steroid injected into the carpal tunnel in patients with moderately severe idiopathic CTS.

**Trial registration:**

Clinicaltrials.gov identifier NCT00806871

## Background

Carpal tunnel syndrome (CTS) is the most common peripheral compression neuropathy in the upper extremity with a prevalence of 3.7% in the adult general population [1]. The prevalence of persons who have moderate or severe CTS but have not sought care or been diagnosed correctly is almost 1% in the general population [2]. CTS of mild severity is commonly treated with wrist splint but for moderately severe symptoms surgical treatment is often required [3]. Carpal tunnel release is one of the most common surgical procedures; the annual incidence in a US population (2001-2005) was 134 per 100,000 [4]

Although carpal tunnel release has been shown to produce good outcomes regarding relief of symptoms caused by CTS [5], it has several disadvantages including surgery-related pain and hand weakness [6]. These are common problems and may last several months after surgery. Other less common complications include wound infection, chronic regional pain syndrome, and nerve injuries [7,8]. In addition, surgery is associated with direct costs as well as indirect costs related to work absence after surgery because the majority of patients require sick leave for a varying length of time depending on the degree of postoperative morbidity and the type of work [9]. The median time of work absence has been 4 weeks in several studies, with a proportion of patients having long-term work disability [9]. The economic impact of work absence following surgery for CTS can be substantial both for the patients and the society. Consequently avoiding sick leave would be an important advantage for nonsurgical treatment.

Although many alternatives to surgery have been proposed there is little evidence to support the efficacy of most of these treatments [10]. Steroid injection into or proximal to the carpal tunnel is widely practiced in the treatment of patients with idiopathic CTS particularly in the United States [11]. However, the evidence for its efficacy beyond one month has not been established in placebo-controlled trials. In a recent Cochrane review of local corticosteroid injection for CTS, the authors concluded that there was evidence supporting clinical improvement at one month compared to placebo but no significant improvement after 8 weeks compared to nonsteroidal anti-inflammatory drugs or wrist splint [12]. None of the studies reviewed involved a double-blind comparison with a follow-up of more than one month.

Because of the strong evidence supporting the efficacy of surgery, randomized studies that compared steroid injections with surgery may provide indirect measure of the efficacy of steroid injection. One such study of 163 wrists comparing surgery with steroid injection (84% received 2 injections within 2 weeks) reported similar effects up to 1 year but outcomes were not assessed with validated CTS-specific measures and it did not compare the full effect size of surgery with that of steroid injection as the primary outcome but rather the proportion of patients in each group that achieved modest improvement [13]. Previous studies comparing steroid injection with surgery have not specifically involved patients whose CTS was of such severity that surgery was clearly indicated. Obviously, if the study population includes many patients with less severe CTS the comparison may be biased. Because the main effects of treatment are improvements in symptoms and in hand function it is important that these outcomes be evaluated with reliable and valid measures [14,15]. The use of steroid injection as a routine treatment in patients with CTS would incur costs and, if ineffective, would prolong patients' disability and delay surgery that usually results in rapid symptom improvement.

In patients with CTS secondary to rheumatoid arthritis the use of steroid injection is based on the assumption that flexor tenosynovitis is the causative factor. This is probably the theoretical basis for using steroid injection even in idiopathic CTS although no such etiological relationship has been established. To our knowledge, the efficacy of local steroid injection in CTS has not been investigated in randomized trials with regard to the possible presence of a dose-response relationship.

There is currently no evidence to support the efficacy of carpal tunnel steroid injection in relieving symptoms of idiopathic CTS for up to 1 year as measured with validated CTS-specific symptom measures and no evidence of possible dose-response relationship.

## Methods/Design

### Design

The trial is a prospective randomized double-blind placebo-controlled dose-response clinical trial comparing injection of 40 mg Methylprednisolone, 80 mg Methylprednisolone or placebo into the carpal tunnel in patients with moderately severe idiopathic CTS not previously treated with steroid injection.

### Inclusion Criteria

The inclusion criteria (Table 1) are primary, idiopathic CTS, age 18 to 70 years, either gender, symptoms of classic or probable CTS according to the diagnostic criteria in the Katz hand diagram [16], symptom duration of at least 3 months, inadequate response to wrist splint, nerve conduction tests showing median neuropathy at the wrist and no other abnormalities or, in the presence of normal nerve conduction test results, two orthopedic surgeons independently diagnose the patient with CTS, and symptom severity of such magnitude that the patient is willing to undergo surgery.

**Table 1:** Eligibility criteria

Inclusion criteria	Exclusion criteria
Primary, idiopathic CTS	Previous steroid injection in same wrist
Age 18-70 years	Inflammatory joint disease
Either gender	Diabetes mellitus
Classic or probable CTS according to the diagnostic criteria in the Katz hand diagram	Polyneuropathy
Symptom duration of at least 3 months	Vibration induced neuropathy
Inadequate response to wrist splint	Pregnancy
Abnormal nerve conduction tests or, if normal, CTS diagnosed independently by 2 surgeons	Trauma to affected hand in preceding year
Patient willing to undergo surgery	Previous surgery for CTS on affected hand
	Surgery for CTS on contralateral hand in preceding 2 months
	Inability to respond to questionnaires
	Severe sensory loss (two-point discrimination >8 mm)
	Thenar atrophy
	Severe medical illness
	Drug or alcohol abuse

### Exclusion Criteria

The exclusion criteria are previous steroid injection for CTS in the same wrist, severe sensory loss (two-point discrimination exceeding 8 mm), thenar atrophy, inflammatory joint disease, diabetes mellitus, vibration-induced neuropathy, polyneuropathy, pregnancy, trauma to the affected hand in the previous year, previous surgery for CTS in the affected hand, surgery for CTS in the contralateral hand within the past 2 months, inability to respond to questionnaires (e.g., because of language difficulty or cognitive impairment), severe medical illness, and known abuse of drugs or alcohol.

### Recruitment

Patients referred by primary care physicians to one orthopedic department (Hässleholm and Kristianstad Hospitals) are examined by trial investigators (orthopedic surgeons) at the outpatient clinic and screened for eligibility. Those with a clinical diagnosis of CTS who have failed treatment with wrist splint and whose symptom severity is judged to warrant surgery are offered to be put on the waiting list for carpal tunnel release. Patients who are willing to undergo surgery are put on the waiting list, which means a waiting time of approximately 3 months. The trial investigator explains the aims, advantages and disadvantages of the trial to eligible patients at the orthopedic department's outpatient clinic. Patients who give informed consent will be allocated to one of the three trial groups. Recruited patients are assigned to a treatment group at the outpatient clinic according to a computer-generated randomization list. Immediately following the allocation the patients will receive the assigned treatment. In patients with bilateral symptoms the most symptomatic hand (as reported by the patient) will be included and in case both hands are equally symptomatic the dominant hand will be included. Patients will not be allowed to enter the trial more than once.

Recruitment to the trial according to the plan described above is believed to ensure the inclusion of a representative sample from the patient population residing in the catchment area. All patients are asked for informed consent before any study procedure is performed. The patients will be recorded in a screening/inclusion/randomization list.

### Ethics

This trial has been approved by the Ethics Committee at the Faculty of Medicine, Lund University (Nr 119-2008) and the Swedish Medical Products Agency (EU 2008-001871-31). The trial will be conducted in full compliance with the Helsinki Declaration.

### Randomization

A computer-generated randomization list of 120 items in blocks of variable sizes (unknown to the investigators involved in recruiting patients) is prepared by the statistician. Based on the list sequentially numbered sealed opaque envelopes containing cards with group assignments are prepared. These sealed envelopes marked with the patients' sequential numbers are kept at the orthopedic department's outpatient clinic. When a patient is enrolled and written informed consent obtained, the nurse opens the envelope with the lowest number and prepares the injection according to the card and then sign date, name and sequential number on the medication list. The nurse preparing the drug is responsible for putting the card in a new envelope marked with the sequential number. This card will be kept in a box with the study medication. As backup, a sealed envelope with the randomization database is kept, together with the study drugs, in the locked area reserved for drugs at the outpatient department. In case the blinding needs to be broken for a patient, the nurse will open the envelope and extract the information for that patient. The envelope will be signed with name and date and given to a monitor.

### Interventions

Group A: 1 ml 40 mg Methylprednisolone (Depomedrol 40 mg/ml) + 1 ml 10 mg Lidocaine (Xylocain 10 mg/ml) + 1 ml saline (NaCl Bayer)

Group B: 2 ml 80 mg Methylprednisolone (Depomedrol 40 mg/ml) + 1 ml 10 mg Lidocaine (Xylocain 10 mg/ml)

Group C: 1 ml 10 mg Lidocaine (Xylocain 10 mg/ml) + 2 ml saline (NaCl Bayer)

The mixture of drugs is prepared by the nurse in the outpatient orthopedic department. This mixture of drugs is standard procedure for Methylprednisolone injection and all nurses have experience in performing this task. The drugs will be injected subfascially in the soft tissue of the carpal canal once by the investigator at baseline according to a previously described injection technique [17]. After injection, patients are instructed to use their hands normally as tolerated; no instructions are given regarding specific activity modifications or splint use.

### Evaluations

At baseline and the last follow-up (Table 2) median nerve conduction testing of both hands is performed. The measurements include median nerve motor distal latency and sensory conduction velocity across the carpal tunnel, median nerve index finger-wrist sensory latency, or ring finger-wrist median-ulnar sensory latency difference.

**Table 2:** Patient visits in the trial, data collection and outcome measures

	Pre-screen	Baseline	5 wk +5 d	10 wk ± 7 d	12 wk^† ^± 7 d	24 wk ± 7 d	52 wk ± 7 d
Diagnosis, inclusion & exclusion criteria	X						
Demographics		X					
Randomization		X					
Physical examination*		X		X		X	X
Nerve conduction testing		X					X
Outcome questionnaires		X	X	X		X	X
Rate of surgery					X	X	X
Sick-leave report		X	X	X		X	X
Adverse events		X	X	X		X	
Study medication		X					
Concomitant medication		X	X	X		X	

*Two-point discrimination, monofilament test, grip and pinch strength

^†^Surgery in participants choosing to have surgery

At baseline and at three follow-up occasions a physical examination is performed by the same physical therapist with extensive experience in hand therapy. Semmes-Weinstein monofilament and two-point discrimination tests of sensation are performed on the radial and ulnar aspects of each finger; two-point discrimination testing starts with a distance of 4 mm that is successively increased by 2 mm until the correct tactile discrimination is recorded. Grip strength and 3-point pinch strength are measured with the Baseline dynamometer and pinch gauge (Chattanooga Group, Hixson, Tennessee, USA), respectively (three trials for each hand). Before examination, the patients are instructed not to discuss their treatment with the assessor and will have their palms covered with a dressing to conceal possible scar after carpal tunnel release. The assessor is thus blinded to whether the patient had or had not undergone surgery after the injection. At the 10-week examination the success of blinding is evaluated by asking the examiner and patient to record whether they think the patient had received active treatment. After the 52-week physical examination the patient will proceed to nerve conduction testing keeping the same palm dressing so that the examiner also will be blinded.

### Follow-up

At the *Baseline *visit, demographics, earlier and ongoing disease, and concomitant medication are recorded. The baseline data include weight, height, and smoking and employment status. The patient completes a questionnaire that includes demographic data, the CTS symptoms severity scale, the 11-item disabilities of the arm, shoulder and hand (*Quick*DASH) questionnaire, and the SF-6D. A physical examination is performed.

At *5 weeks *after injection a telephone interview is conducted by the investigator and the patient is asked to complete the CTS symptoms severity scale and concomitant medication and adverse events (AEs) will be recorded.

At *10 weeks *after injection the patient is examined by the physical therapist. The investigator asks the patient whether she/he wants to proceed with surgery as planned at 12 weeks after injection or whether the patient has experienced adequate symptom relief that she/he wants to continue follow-up and not have the planned surgery at this stage. An evaluation similar to the baseline evaluation is performed and the indication for surgery is recorded. The patient completes the CTS symptoms severity scale. Concomitant medication and AEs are recorded. Possible use of a wrist splint since the injection and its frequency is recorded.

At 12 weeks the patient who had chosen to have surgery is operated on as scheduled.

At *24 weeks *after injection the patient is examined and asked to complete a questionnaire including the CTS symptoms severity scale, *Quick*DASH, and the SF-6D as well as to rate satisfaction with the treatment on a visual analog scale from 0 (very dissatisfied) to 100 (completely satisfied). Concomitant medication and AEs are recorded.

At *52 weeks *after injection the patient is asked to attend a physical examination and nerve conduction tests and complete the questionnaires.

### Outcome Measures

The CTS symptoms severity scale is a validated 11-item questionnaire that mainly inquires about severity, frequency and duration of night and daytime pain and numbness or tingling [14,15]. The CTS score ranges from 1 (no symptoms) to 5 (most severe symptoms). The *Quick*DASH is a validated 11-item questionnaire that mainly inquires about difficulties in performing daily activities, yielding a score from 0 (no disability) to 100 (worst possible disability) [18]. The SF-6D is a validated health utility measure that is used to compare cost-effectiveness of different treatments and the value ranges from -0.11 (worst health) to 1.0 (perfect health) [19]. All AEs are recorded during 24 weeks. Duration of sick leave for employed patients is recorded.

### Blinding

The nurse at the outpatient clinic prepares the medication with a label covering the syringe. The injection will then be administered to the patient by the investigator. The placebo is clear and Methylprednisolone is a white suspension. In subfascial injections such as in the carpal canal leakage from the injection canal is small. The investigator administering the injection presses a medical dressing over the puncture site while withdrawing the needle; this will conceal the color in case of leakage. The decision whether or not to have surgery is done by the patient at 10 weeks and the patients are instructed to make that choice based on their symptoms and functional status at that time. At the 24-week and 52-week patient visits the palm is covered by a bandage with a dressing to blind the examining physical therapist as to whether the patient had undergone surgery after the injection. All follow-up evaluations are done by a physical therapist blinded to the group assignment.

### Sample Size

Based on previous studies of surgical outcome in patients with idiopathic CTS the mean improvement in the CTS symptom severity scale at 12 weeks after surgery is about 1.6 (on the 1 to 5 point scale). A mean improvement of less than 0.8 in the CTS symptom severity score cannot warrant recommending steroid injection as an effective alternative treatment. With 90% statistical power, 5% significance level, and two-sided statistical tests, and assuming a standard deviation of 1.0 for the CTS symptom severity score the study can detect a true difference of at least 0.8 point on the CTS symptom severity score between an intervention group and the placebo group when 102 patients have been randomized. The aim will be to enroll 120 patients in the study to account for potential incomplete follow-ups.

### Primary endpoint

1. The CTS symptom severity score change at 10 weeks after treatment:

1) 80 mg Methylprednisolone vs. placebo

2) 40 mg Methylprednisolone vs. placebo

3) 80 mg Methylprednisolone vs. 40 mg Methylprednisolone

2. The rate of surgery at (in rank order) 52 weeks, 24 weeks, and 12 weeks after treatment:

1) 80 mg Methylprednisolone vs. placebo

2) 40 mg Methylprednisolone vs. placebo

3) 80 mg Methylprednisolone vs. 40 mg Methylprednisolone

### Secondary endpoint

1. The CTS symptom severity score change at 52 weeks

2. Time to surgery

3. The *Quick*DASH score at 10 weeks and 52 weeks

4. The SF-6D score at 10 weeks and 52 weeks

5. Patient satisfaction with treatment at 10 weeks and 52 weeks

6. Duration of sick leave at 52 weeks (for employed patients)

### Statistical Analysis

All patients who have been randomized and have received an injection will be included in the statistical evaluation. Statistical tests will be performed and reported according to the intention-to-treat principle. Data will be presented as mean and standard deviation or median and range, as appropriate, for continuous variables and as numbers and proportions for categorical variables. The primary analysis of the CTS symptom severity score at 10 weeks will be performed using a mixed model analysis of repeated measures (baseline, 5 weeks, 10 weeks) with treatment effect (placebo or Methylprednisolone) and time as fixed factors in a first step and with the interaction term of treatment and time in second step. The results will be presented as differences in mean score change over time (baseline to 10 weeks) and 95% confidence intervals. The secondary analysis of the symptom severity score will be done using a mixed model with treatment (placebo or Methylprednisolone) as a random factor and time (baseline, 5 weeks, 10 weeks, 24 weeks, 52 weeks) as fixed factor and surgery as covariate; first step without interaction terms and second step with two-way interaction of treatment and time. The results will be presented as differences in mean score change over time and 95% confidence intervals.

The primary analysis of rate of surgery will be done using Fisher's exact test (univariate) and logistic regression (multivariate) with adjustment for baseline variables. The issue of multiplicity is addressed by performing the analysis in the following priority; 52 weeks, 24 weeks, and 12 weeks. The results will be presented as differences in the rate of surgery between the groups (Methylprednisolone compared with placebo) and as adjusted odds ratio (and 95% confidence interval) of having surgery at each time point. The possible dose-response relationship with higher effect of high dose will be explored by prioritizing group comparisons. We will conduct the following predefined subgroup analyses; symptom duration (12 months or shorter versus more than 12 months), symptom severity (baseline symptom severity score below 3.0 versus 3.0 or higher), and median nerve conduction abnormality (moderate or severe versus mild or normal).

Analysis of the secondary end-points will be done with mixed models adjusting for baseline variables. We will also calculate the effect size for the symptom and function scales [20]. The AEs will be reported in table form and stratified in 4 groups according to severity; statistical comparison will be done when appropriate. When at least 60 patients have completed the 24-week visit the frequency of SAEs and of AEs with persistent symptoms will be calculated and the results will be reported and discussed with the Swedish Medical Products Agency.

## Discussion

In this double-blind placebo-controlled randomized trial of patients with moderately severe idiopathic CTS we intend to investigate the medium-term efficacy of steroid injection into the carpal tunnel and the possible presence of a dose-response relationship first at 10 weeks but also at 24 weeks and 52 weeks. The strength of this trial is that it will be performed at one orthopedic department to which the vast majority of patients with CTS from the study region are referred. In addition, steroid injection is rarely used as a treatment for primary idiopathic CTS by primary care physicians who usually treat patients with CTS in the study region before referring them to the orthopedic department.

A potential factor that may influence the results is that all patients are given the choice to have surgery at 12 weeks if their symptoms have not improved adequately. Before inclusion in the trial all the patients have unsuccessfully undergone treatment with wrist splinting and have symptoms of such severity that warranted referral to the orthopedic department. Because surgery is the only remaining treatment option with a strong evidence of efficacy there is no ethically acceptable alternative to offering surgery after 12 weeks. This trial will not answer the question of whether steroid injection is effective in patients with CTS that failed wrist splinting but the severity of which is judged by the patient as mild and not warranting surgery or in patients declining surgery for other reasons. The use of the standardized CTS symptom severity scale in the trial will show the severity spectrum of CTS in the study population. Another factor is the potential multiplicity of the analyses but this is addressed by providing a rank order with which the analyses will be carried out.

Our goal is to determine the efficacy of two different doses of local steroid injection in the treatment of moderately severe CTS, taking into account the natural course of CTS and the placebo effect of injection. During the first 24 weeks the safety of steroid injection in the carpal tunnel will also be evaluated in comparison with placebo. The results will also be useful to predict the effect of treatment with steroid injection related to severity of CTS. Information on possible relationship between dosage and duration of effect also will be provided by this study.

## Competing interests

The authors declare that they have no competing interests.

## Authors' contributions

MF and IA drafted the manuscript. MF wrote the original protocol with guidance and revision by IA, secured funding together with IA and will be responsible for subject recruitment and treatment during the trial and the overall management of the trial. IA designed the study, guided the development of the study protocol and secured funding. MH participated in the planning and conduction of the study. JB was involved in developing the statistical layout of the study. All authors have contributed to and approved the final version of this manuscript.

## Pre-publication history

The pre-publication history for this paper can be accessed here:

http://www.biomedcentral.com/1471-2474/11/76/prepub
